# Investigation of image plane for image reconstruction of objects through diffusers via deep learning

**DOI:** 10.1117/1.JBO.27.5.056001

**Published:** 2022-05-04

**Authors:** Takumi Tsukada, Wataru Watanabe

**Affiliations:** Ritsumeikan University, College of Science and Engineering, Department of Electrical and Electronic Engineering, Kusatsu, Shiga, Japan

**Keywords:** deep learning, scattering imaging, diffuser, speckle

## Abstract

**Significance:**

The imaging of objects hidden in light-scattering media is a vital practical task in a wide range of applications, including biological imaging. Deep-learning-based methods have been used to reconstruct images behind scattering media under complex scattering conditions, but improvements in the quality of the reconstructed images are required.

**Aim:**

To investigate the effect of image plane on the accuracy of reconstructed images.

**Approach:**

Light reflected from an object passing through glass diffusers is captured by changing the image plane of an optical imaging system. Images are reconstructed by deep learning, and evaluated in terms of structural similarity index measure, classification accuracy of digital images, and training and testing error curves.

**Results:**

The reconstruction accuracy was improved for the case in which the diffuser was imaged, compared to the case where the object was imaged. The training and testing error curves show that the loss converged to lower values in fewer epochs when the diffuser was imaged.

**Conclusions:**

The proposed approach demonstrates an improvement in the accuracy of the reconstruction of objects hidden through glass diffusers by imaging glass diffuser surfaces, and can be applied to objects at unknown locations in a scattering medium.

## 1 Introduction

Random media or scattering media such as frosted glasses and biological tissue significantly change and diffuse the properties of transmitted light, which reduces the quality of transmitted images. Visualization of objects inside or through scattering media is a challenging problem for various applications. In recent years, a variety of approaches such as point spread functions,^1^^,^^2^ speckle correlation,^3^ transmission matrices,^4^ and wavefront shaping^5^^,^^6^ have been demonstrated to visualize objects behind scatterers using randomly scattered light. However, these imaging methods do not perform well for objects obscured by complex scattering media.^7^ Recently, data-driven approaches to imaging through scattering media have been proposed using support vector regression architecture^8^ and deep learning.^9^ Deep-learning-based methods solve inverse problems by introducing a large number of pairs of object images and speckle images to the deep-learning model.^10^^,^^11^ The model trains an inverse scattering function, which enables the reconstruction of object images.^12^ Various methods have been proposed for training networks. Among these networks, convolutional neural networks (CNNs) have been used as a more flexible and generalized technique to reconstruct objects behind scatterers or diffusers. CNNs have been used to retrieve amplitude objects behind a random medium using either a single^13^ or multiple diffusers.^14^ Deep-learning-based methods also enable imaging under low-photon conditions^15^ and reconstruction of objects through dynamic scattering media.^16^ However, these methods face several challenges, such as the selection of the training framework, scalability, and optical systems. The scope of this work is limited to optical systems for imaging through light-scattering obscurants using deep learning. Optical systems can be classified into those encoding objects as transmission mode^8^^,^^14^^,^^15^^,^^17^^–^^19^ and reflection mode.^9^^–^^11^^,^^13^^,^^16^^,^^20^^–^^24^ In both modes, optical systems, such as lensless imaging systems,^8^^,^^10^^,^^17^^,^^20^^,^^21^^,^^23^ imaging systems with one or two lenses,^9^^,^^11^^,^^14^^–^^16^^,^^18^^,^^19^^,^^22^ and imaging systems with a camera lens^13^^,^^24^ have been investigated. Among these, the 4f optical system consisting of two lenses has the advantage of removing scattered light by spatial filtering, a large field of view, ease of magnification by replacing the lens system, and light-collection efficiency.^9^^,^^11^^,^^18^^,^^19^^,^^22^ In previous reports, the image plane was set as an object behind glass diffusers, assuming that the image plane was known.^9^^,^^11^^,^^18^ In real space, the image plane of an object is unknown when the object is hidden by scattering materials or glass diffusers. In addition, when objects are located in thick scattering media, such as biomedical tissue, image reconstruction of objects at unknown depths must be considered.

In this study, we investigate the effects of the image plane of an optical system on the classification accuracy of reconstructed images through glass diffusers using deep learning. We investigate two cases, one in which the diffuser is imaged and the other where the object is imaged. The experimental results show that imaging on the diffuser plane improves the accuracy compared to imaging the object plane through the image reconstruction and image evaluation.

## 2 Method

**Fig. 1:**
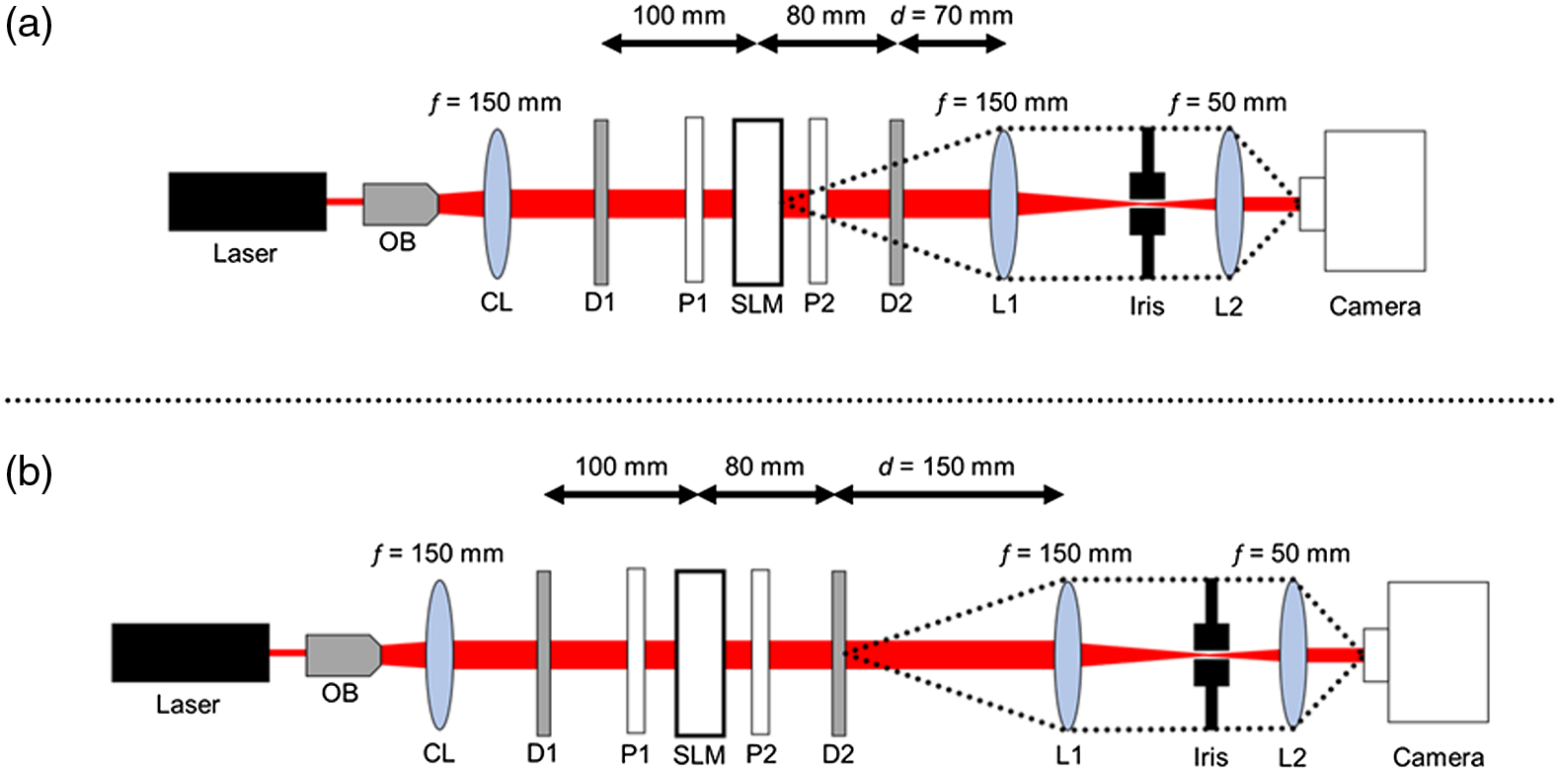
Schematic of imaging system for acquiring speckle images. OB: objective lens, CL: collimating lens, D: diffuser, P: polarizer, SLM: spatial light modulator, and L1, L2: lens. The distance between the diffuser D2 and the lens L1 was denoted by d. (a) SLM plane is imaged. (b) D2 plane is imaged.

A schematic of the optical system is shown in Fig. 1. We used a transmission-type spatial light modulator (SLM) (Holoeye LC2012, 36-μm pixel pitch, 1024×768). To obtain amplitude modulation, we placed a polarizer P2 whose polarization direction was perpendicular with respect to P1. The central 160×160 pixels of the SLM were used in the experiments. Coherent light from a He–Ne laser source (LASOS LGK7654-8, 632.8 nm) was expanded with an objective lens (OB) with a 20× magnification, and was collimated by the collimating lens (CL) (f=150  mm). The 4f optical system consisted of two lenses L1 (f=150  mm) and L2 (f=50  mm). Speckle images were recorded with a charged coupled device (CCD) camera (The Imaging Source DMK23U445, 8 bits, 3.75-μm pixel pitch, 1280×960), of which only the central 256×256 portion was used in the experiments. In Fig. 1(a), the object plane on the SLM was imaged on the CCD camera. In Fig. 1(b), the diffuser plane (D2) was on the CCD camera. The distance between diffuser D1 and SLM was set to 100 mm, and that between the SLM and diffuser D2 was set to 80 mm. An iris with a diameter of 7.5 mm was placed at the Fourier plane of the lens L1.

To evaluate our approach, we used the MNIST dataset of handwritten digits as input images for SLM.^25^ For diffusers D1 and D2, we used a holographic diffuser and a white diffusive glass, as shown in Table 1.

**Table 1:** Description of diffusers used in experiments.

Name	Diffusion angle	Thickness
Holographic diffuser (Edmund Optics 47996)	5 deg	0.78 mm
White diffusing glass (Edmund Optics 34480)	120 deg	1.25±0.1 mm

**Fig. 2:**
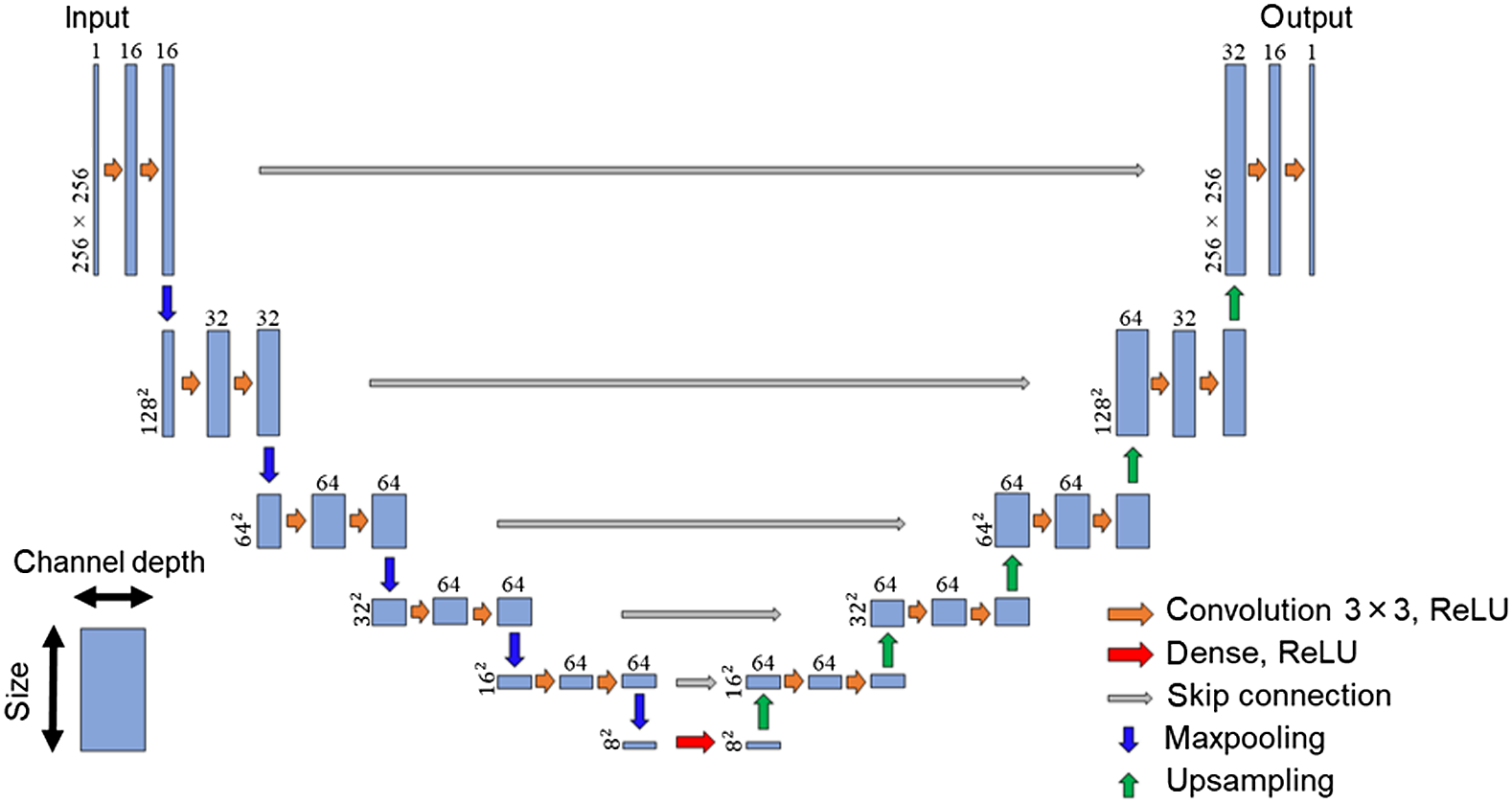
The deep-learning model used for image reconstruction. The structure of the deep-learning model follows the U-Net architecture. The height of each block represents the size. The width of each block represents the channel depth.

The deep-learning model used in this experiment is shown in Fig. 2. We used CNN, and our deep-learning model possessed a U-Net architecture.^26^ The model was trained using speckle images of 256×256×1 (256×256 xy size, 1 channel depth) acquired by the camera as inputs. In the encoding part, the input data were repeatedly passed through two convolutional layers with a 3×3 kernel and a maxpooling layer with a 2×2 kernel. The xy size was reduced and the channel depth increased. Finally, the input data resulted in 8×8×64 (8×8 xy size, 64 channel depth). The encoding part and the decoding part were connected with a densely connected layer. In the decoding part, the channel depth was gradually reduced back to the same size as the input size, in contrast to the encoding part. Only the convolution layer before the output had a kernel size of 1×1 to match the output size. The loss function was the negative Pearson correlation coefficient (NPCC), and we used Adam to update the weights.^11^ NPCC is expressed as

$$\mathrm{NPCC}=\frac{-1\times \sum_{i=1}^{w}\sum_{j=1}^{h}(X(i,j)-\overset{¯}{X})(Y(i,j)-\overset{¯}{Y})}{\sqrt{\sum_{i=1}^{w}\sum_{j=1}^{h}(X(i,j)-\overset{¯}{X}{)}^{2}}\sqrt{\sum_{i=1}^{w}\sum_{j=1}^{h}(Y(i,j)-\overset{¯}{Y}{)}^{2}}},$$ (1)

where w and h are the vertical and horizonal sizes of the images, X(i,j) and Y(i,j) are ground-truth images and reconstructed images, respectively, and $\overset{¯}{X}$ and $\overset{¯}{Y}$ are the mean values of pixels in these images. Keras was used to build the deep-learning model, which was run on a GPU (GeForce GTX 1660 Ti).

We captured 3000 speckle images, of which 2220 were used for training, 560 for validation, and 200 for testing. We used structural similarity (SSIM) and accuracy (ACC) as indices of evaluation of the reconstructed images. ACC denotes the classification accuracy of the digital images, which is defined by the correct answer rates of identification in the number recognition program trained by CNN. SSIM is calculated as

$$\mathrm{SSIM}(x,y)=\frac{\left(2\mu_{x}\mu_{y}+C_{1})(2\sigma_{xy}+C_{2}\right)}{\left(\mu_{x}^{2}+\mu_{y}^{2}+C_{1})(\sigma_{x}^{2}+\sigma_{y}^{2}+C_{2}\right)},$$ (2)

where $\mu_{x}$ and $\mu_{y}$ are the means of the pixel value, $\sigma_{x}^{2}$ and $\sigma_{y}^{2}$ are the variances of the pixel values, $\sigma_{xy}$ is the covariance of the pixel values, $C_{1}=(255\times 0.01{)}^{2}$ and $C_{2}=(255\times 0.03{)}^{2}$ are the normalization constants. The SSIM and ACC in the following results are the average values of 200 reconstructed images.

## 3 Experimental Results and Discussion

### 3.1 Image Reconstruction Using One Diffuser

**Fig. 3:**
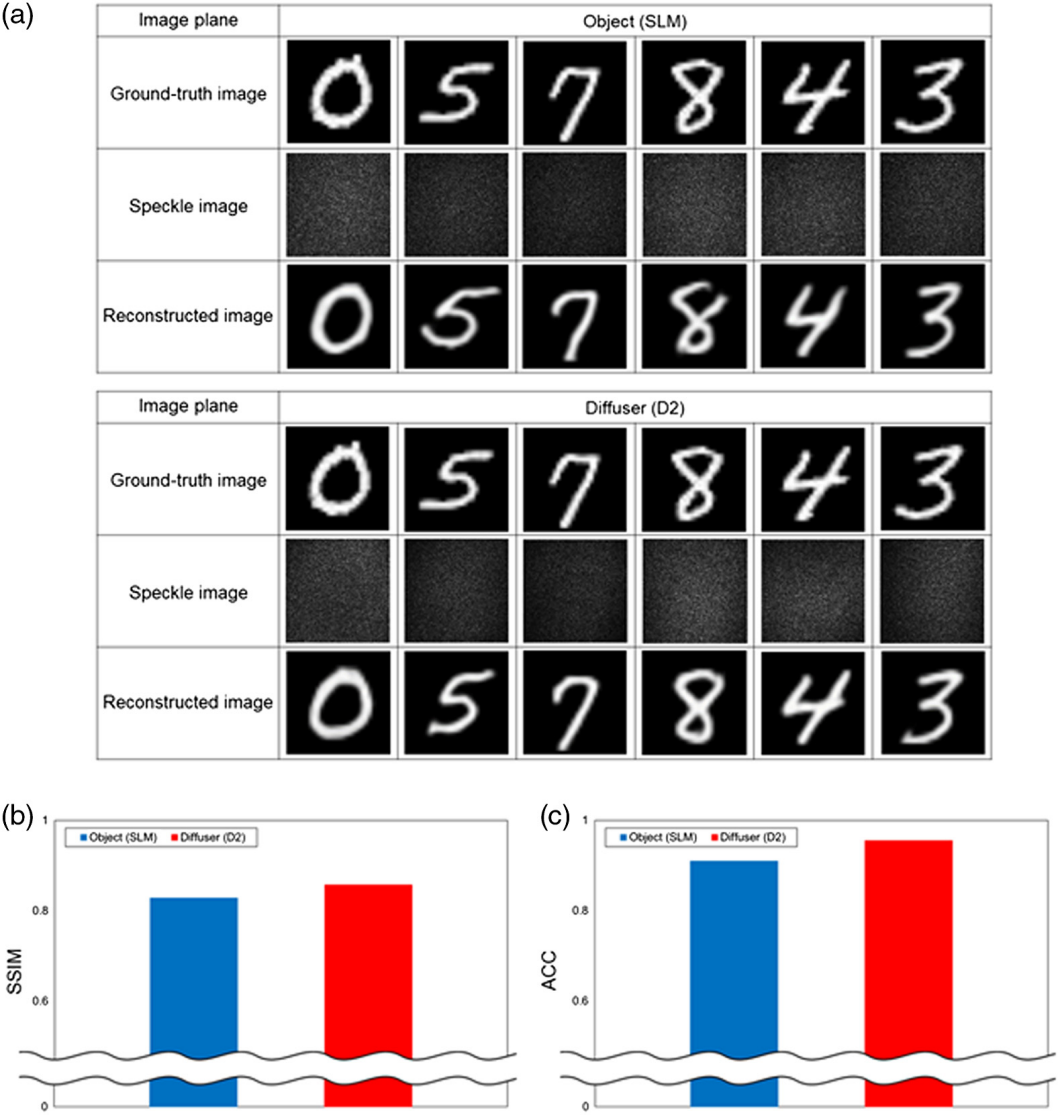
(a) Reconstruction results of objects behind the diffuser and (b) SSIM and (c) ACC evaluations of reconstructed images. The legends correspond to the image planes.

To investigate the image plane in the optical system, we first conducted experiments using one diffuser. We only used the white diffusive glass with a diffusion angle of 120 deg as D2 and no diffuser was placed at D1, as shown in Figs. 1(a) and 1(b). Figure 3 shows the reconstruction results and the evaluations of the reconstructed images. The images were reconstructed as high-quality images in both image planes, as shown in Fig. 3(a). Figures 3(b) and 3(c) show the SSIM and ACC evaluations, respectively. As shown in Fig. 3(b), the SSIM was ∼0.828 when the object was imaged and ∼0.857 when the diffuser was imaged. As shown in Fig. 3(c), the ACC was 91% when the object was imaged and 95.5% when the diffuser was imaged. Evaluations of the reconstructed images were better when the image plane was set as the diffuser and one diffuser was placed between the object and collecting lens L1 in a 4f imaging system.

### 3.2 Image Reconstruction of an Object Placed between Two Diffusers

**Fig. 4:**
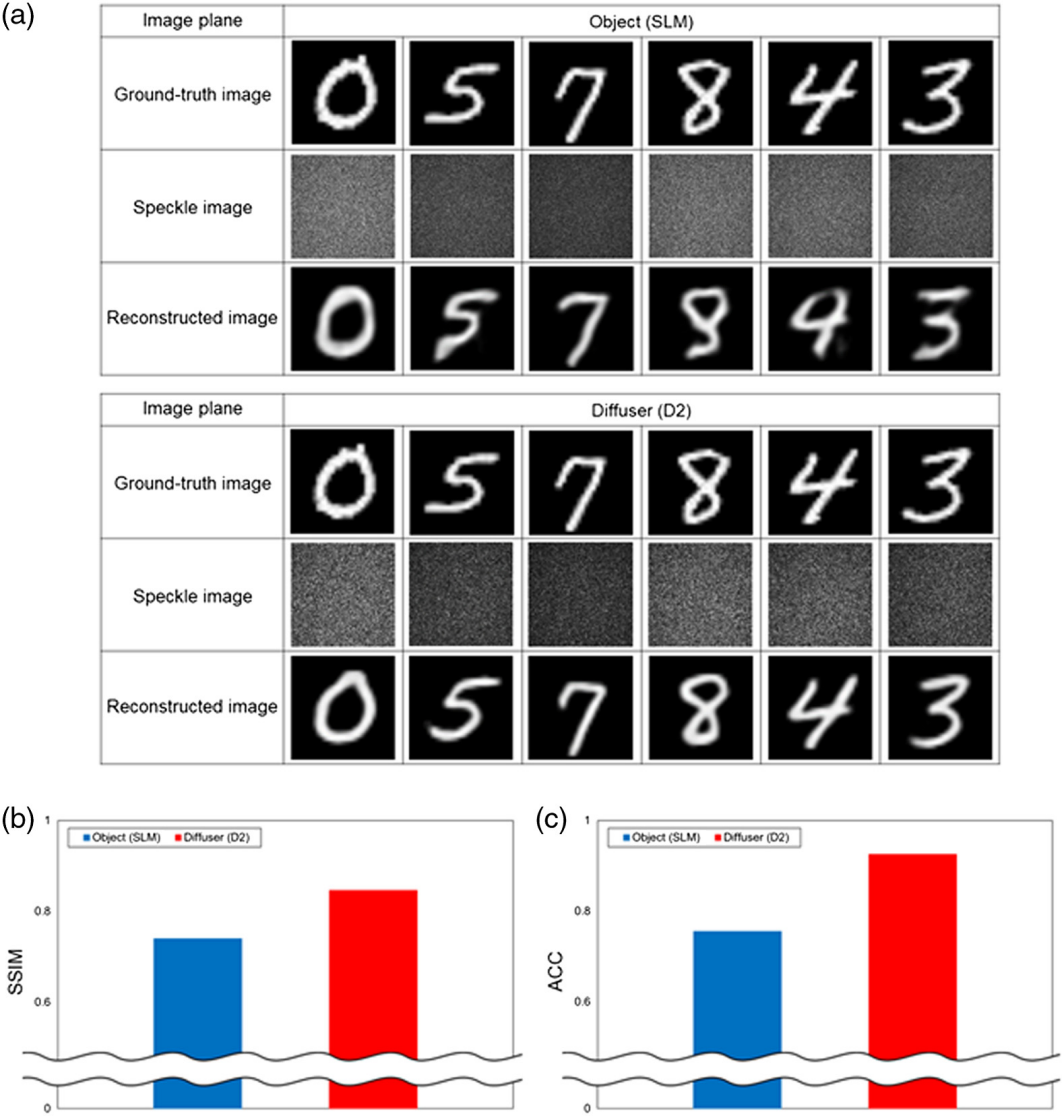
(a) Reconstruction results of objects placed between two diffusers and (b) SSIM and (c) ACC evaluations of reconstructed images.

A difficulty arises in imaging an object placed between two diffusers, in that the object is illuminated with a diffused wave.^14^^,^^27^^–^^29^ We investigated image reconstruction of an object placed between two diffusers, as shown in Fig. 1. We used the holographic diffuser as D1 and white diffusive glass as D2. Figure 4 shows the reconstruction results and the evaluations of the reconstructed images. As shown in Fig. 4(a), when the object was imaged, the reconstructed images were deformed. However, when the diffuser was imaged, the reconstructed images were of high quality even when the object was placed between the two diffusers. Figures 4(b) and 4(c) show the SSIM and ACC evaluations, respectively. As shown in Fig. 4(b), the SSIM was ∼0.739 when the object was imaged and ∼0.846 when the diffuser was imaged. As shown in Fig. 4(c), the ACC was 75.5% when the object was imaged and 92.5% when the diffuser was imaged. Based on the results, it may be noted that the accuracy was higher when the diffuser was imaged than when the object behind the diffuser was imaged.

**Fig. 5:**
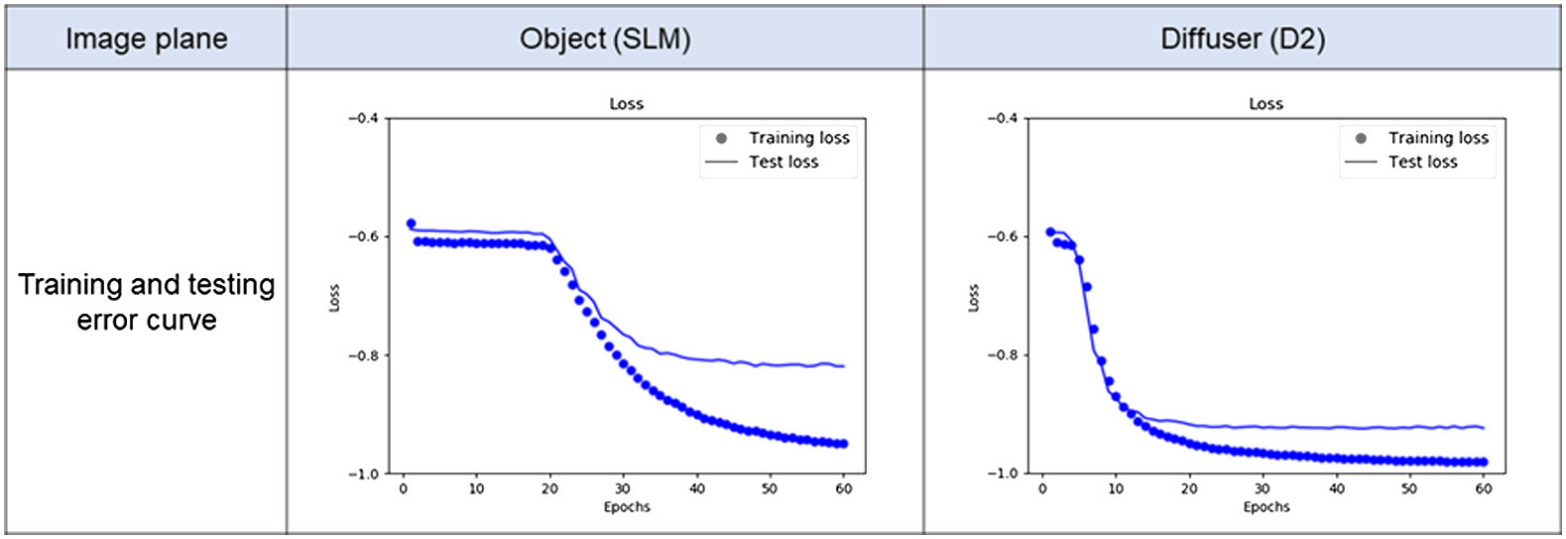
Quantitative analysis with the training and testing error curves. Training and testing error of CNN using NPCC as the loss function.

The training and testing error curves of the CNN for each imaging condition are shown in Fig. 5, for the experimental conditions where the object was placed between two diffusers. The left panel in Fig. 5 shows the dependence of the loss value on the epoch number when the object was imaged, showing that the test loss value decreased with the epoch number and eventually reached a minimum −0.81 in the training process of the CNN. The test loss converged at ∼40 epochs, and the difference between the training loss and test loss was large. The right panel in Fig. 5 shows the dependence of the loss value on the epoch number when the diffuser was imaged, showing that the loss value reached a minimum of −0.92 in the training process of the CNN. The testing loss converged faster, i.e., epochs of ∼20 epochs, and the difference between the training loss and test loss was smaller than that when the object was imaged.

**Fig. 6:**
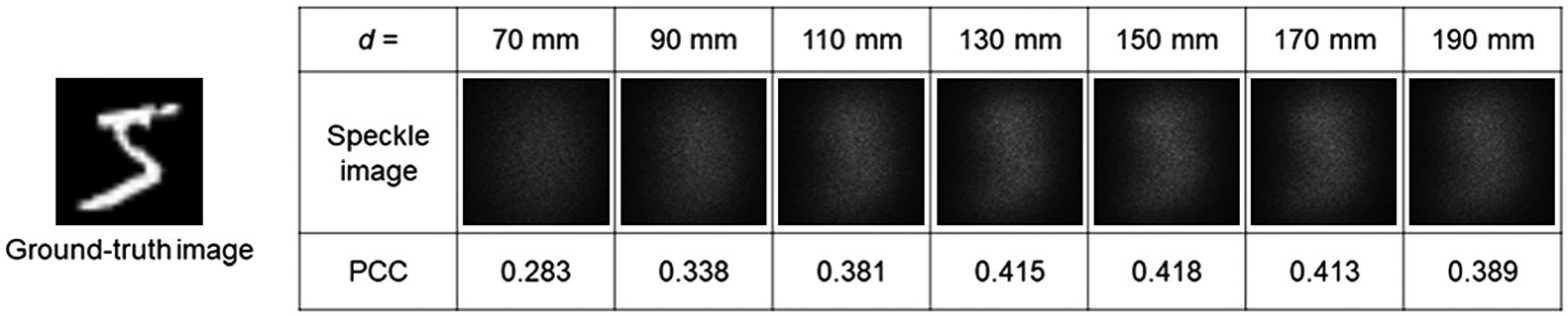
The PCC between ground-truth image and speckle images captured by changing the image plane.

To gain insight into the dependence of the quality on the image plane, we calculated the Pearson correlation coefficient (PCC) between the ground-truth image and speckle images at different image planes by changing the distance between the diffuser D2 and the lens L1, d (see Fig. 1). Figure 6 shows the PCC between ground-truth image and speckle images captured by changing the image plane. When d was 70 mm, the SLM was imaged [Fig. 1(a)]. When d was 150 mm, the diffuser D2 was imaged [Fig. 1(b)]. The PCC became higher as the image plane approached d=150  mm, and the highest PCC was obtained at the distance d of 150 mm. When the image plane was defocused from the diffuser D2 to the camera side, the PCC decreased. When the object was imaged, the propagated and diffracted light from the object was diffused by a diffuser. Therefore, imaging the object was associated with more complex training and reconstruction processes. When the diffuser was imaged, the propagation and diffracted light from the object is projected onto the diffuser. The diffuser plane is considered to be a secondary image plane by mapping the plane of the object image; therefore, the training becomes simple. The image plane affects the image reconstruction results and epochs in deep learning.

### 3.3 Discussion

We investigated the dependence of the distance between the SLM and diffuser D2 by shifting D2 along the optical axis when the object was imaged. When the distance between the SLM and D2 increased, the training speed decreased. As mentioned in Sec. 3.2, when the diffuser was placed in front of the object, the diffuser surface becomes the secondary image plane. Therefore, when the object was imaged, the closer the distance between the object (SLM) and the diffuser (D2), the smaller the effect of defocus and the faster the training speed. When the diffuser was imaged, the accuracy was better than when the object was imaged because the effect of defocus was the least.

The position of the two diffusers in Sec. 3.2 was switched, and the experiment was also conducted with the white diffusive glass (diffusion angle of 120 deg) as D1 and the holographic diffuser (diffusion angle of 5 deg) as D2. When the diffusion angle in D2 was smaller, over 90% ACC was achieved in both image plane conditions. The results show that the diffuser placed behind the object, D2, had a strong effect on the image reconstruction. The diffusion angle in D2 was smaller, and the reconstruction accuracy improved.

We performed experiments with two diffusers placed in front of the object with respect to lens L1. In this case, no diffuser was placed at D1, as shown in Fig. 1, and two diffusers were placed between the object and lens L1. The reconstruction accuracy was also better when the diffusers were imaged than when the object was imaged, and more than 90% of the ACC was achieved by imaging the diffusers.

In these experiments, we demonstrated the feasibility of our approach by using diffusers with diffusion angles of 5 deg and 120 deg. Similar trends were obtained with the combination of two diffusers having diffusion angles of 5 deg, 10 deg, 20 deg, and 120 deg.

We verified our approach by using a 4f optical system. This approach is versatile, being feasible with different optical systems. We performed experiments using a camera with a C-mount lens, which has a wide field of view. In this experiment, L1, L2, and the iris shown in Fig. 1 were removed, and a C-mount lens (The Imaging Source TCL 1216 5MP) was directly attached to the CCD camera. The image plane was set to be the diffuser surface, and the speckle images were captured by a camera with a C-mount lens with two diffusers. We used a holographic diffuser with a diffusion angle of 5 deg as D1 and white diffusive glass on D2. The results demonstrated that over 90% ACC was achieved.

## 4 Conclusion

We have demonstrated an imaging technique that allows the reconstruction of objects hidden behind glass diffusers by investigating their image planes in optical imaging systems. We reconstructed an object behind a diffuser or placed between two diffusers in a 4f optical system with deep learning. The accuracy improved when the diffuser was imaged. By imaging the diffuser, scattering imaging can be performed regardless of the position of the object behind the diffuser. These results are expected to be applied to cases where the target object is located inside a scattering medium with an unknown position, such as in biological imaging.
